# Preoperative Prediction of Axillary Lymph Node Metastasis in Breast Cancer Using Radiomics Features of Voxel-Wise DCE-MRI Time-Intensity-Curve Profile Maps

**DOI:** 10.3390/biomedicines13102562

**Published:** 2025-10-21

**Authors:** Ya Ren, Kexin Chen, Meng Wang, Jie Wen, Sha Feng, Honghong Luo, Cuiju He, Yuan Guo, Dehong Luo, Xin Liu, Dong Liang, Hairong Zheng, Na Zhang, Zhou Liu

**Affiliations:** 1 Department of Radiology, National Cancer Center/National Clinical Research Center for Cancer/Cancer Hospital & Shenzhen Hospital, Chinese Academy of Medical Sciences and Peking Union Medical College, Shenzhen 518116, Chinawangmeng@cicams-sz.org.cn (M.W.); 18503083950@sina.cn (J.W.); lhonghong2025@163.com (H.L.); 13926236152@163.com (D.L.); 2 Paul C. Lauterbur Research Center for Biomedical Imaging, Shenzhen Institute of Advanced Technology, Chinese Academy of Sciences, Shenzhen 518055, China; kx.chen2@siat.ac.cn (K.C.); xin.liu@siat.ac.cn (X.L.); dong.liang@siat.ac.cn (D.L.); hr.zheng@siat.ac.cn (H.Z.); 3 Department of Biomedical Engineering, Southern University of Science and Technology, Shenzhen 518055, China; 4 Department of Pathology, National Cancer Center/National Clinical Research Center for Cancer/Cancer Hospital & Shenzhen Hospital, Chinese Academy of Medical Sciences and Peking Union Medical College, Shenzhen 518116, China; fengsha_89@163.com; 5 Liaoning Cancer Hospital & Institute, Shenyang 110042, China; cuijuhe@yeah.net; 6 Department of Radiology, Guangzhou First People’s Hospital, South China University of Technology, Guangzhou 510180, China; eyguoyuan@scut.edu.cn; 7 Key Laboratory of Biomedical Imaging Science and System, Chinese Academy of Sciences, Shenzhen 518055, China

**Keywords:** axillary lymph node metastasis, dynamic contrast-enhanced MRI (DCE-MRI), time-intensity curve (TIC), radiomics, hemodynamic heterogeneity

## Abstract

**Objective**: Axillary lymph node (ALN) status in breast cancer is pivotal for guiding treatment and determining prognosis. The study aimed to explore the feasibility and efficacy of a radiomics model using voxel-wise dynamic contrast-enhanced magnetic resonance imaging (DCE-MRI) time-intensity-curve (TIC) profile maps to predict ALN metastasis in breast cancer. **Methods**: A total of 615 breast cancer patients who underwent preoperative DCE-MRI from October 2018 to February 2024 were retrospectively enrolled and randomly allocated into training (*n* = 430) and testing (*n* = 185) sets (7:3 ratio). Based on wash-in rate, wash-out enhancement, and wash-out stability, each voxel within manually segmented 3D lesions that were categorized into 1 of 19 TIC subtypes from the DCE-MRI images. Three feature sets were derived: composition ratio (type-19), radiomics features of TIC subtypes (type-19-radiomics), and radiomics features of third-phase DCE-MRI (phase-3-radiomics). Student’s *t*-test and the least absolute shrinkage and selection operator (LASSO) was used to select features. Four models (type-19, type-19-radiomics, type-19-combined, and phase-3-radiomics) were constructed by a support vector machine (SVM) to predict ALN status. Model performance was assessed using sensitivity, specificity, accuracy, F1 score, and area under the curve (AUC). **Results**: The type-19-combined model significantly outperformed the phase-3-radiomics model (AUC = 0.779 vs. 0.698, *p* < 0.001; 0.674 vs. 0.559) and the type-19 model (AUC = 0.779 vs. 0.541, *p* < 0.001; 0.674 vs. 0.435, *p* < 0.001) in cross-validation and independent testing sets. The type-19-radiomics showed significantly better performance than the phase-3-radiomics model (AUC = 0.764 vs. 0.698, *p* = 0.002; 0.657 vs. 0.559, *p* = 0.037) and type-19 model (AUC = 0. 764 vs. 0.541, *p* < 0.001; 0.657 vs. 0.435, *p* < 0.001) in cross-validation and independent testing sets. Among four models, the type-19-combined model achieved the highest AUC (0.779, 0.674) in cross-validation and testing sets. **Conclusions**: Radiomics analysis of voxel-wise DCE-MRI TIC profile maps, simultaneously quantifying temporal and spatial hemodynamic heterogeneity, provides an effective, noninvasive method for predicting ALN metastasis in breast cancer.

## 1. Introduction

Breast cancer is the most prevalent malignancy and the leading cause of tumor-related deaths in females worldwide, significantly impacting women’s health [1]. Accurate preoperative assessment of axillary lymph node (ALN) status of breast cancer is critical, as patients with ALN metastasis exhibit up to 40% lower 5-year survival rates compared to ALN non-metastasis patients [2], significantly influencing treatment planning (surgical extent, neoadjuvant chemotherapy + surgery, or surgery directly) and prognosis [3,4]. Currently, the gold standard for diagnosing the metastasis of ALN is axillary lymph node dissection (ALND), along with sentinel lymph node biopsy (SLNB), yet both are invasive, posing risks like numbness, lymphedema, and infection [5,6]. Furthermore, SLNB also has a false-negative rate between 7.8% to 27.3% [7]. Therefore, there is a pressing need for an accurate and noninvasive method to predict the ALN status in breast cancer patients preoperatively.

Breast dynamic contrast-enhanced magnetic resonance imaging (DCE-MRI), with its favorable soft-tissue contrast and its capacity to capture tumor hemodynamics, is crucial for assessing ALN status. While DCE-MRI-related parameters of primary breast tumors (i.e., Ktrans) have shown promise in predicting ALN metastasis [8,9], the traditional qualitative, semi-quantitative, or model-based quantitative analyses employed each bear inherent limitations. Qualitative analysis is limited by the random selection of regions of interest (ROIs), averaging effects, and observer variability [10]. Semi-quantitative analysis similarly suffers from ROI selection bias, averaging effects, and inconsistencies in definitions [11]. Moreover, model-based quantitative analysis, based on idealized models (e.g., two-compartment models), faces challenges from MRI scan parameters, baseline T1 values of pre-contrast tissue, arterial input functions, and various artifacts, making it challenging to apply in clinical practice [12].

To address these issues, we previously proposed a novel model-free, data-driven method to generate the voxel-wise mapping of diverse DCE-MRI time-intensity-curve (TIC) profiles, facilitating visualization of the intra-tumor temporal hemodynamic heterogeneity. While we demonstrated that the composition ratios of these TIC profiles within 3D lesions effectively distinguish benign from malignant tumors [13], this approach does not quantify the spatial heterogeneity of TIC subtypes.

Radiomics, which extracts high-dimensional features from medical images, quantifies spatial heterogeneity [14,15] and has shown potential in predicting ALN status (AUC: 0.710–0.862) using DCE-MRI features from primary tumors [16,17,18]. However, these studies predominantly focus on radiomics based on a single-phase DCE-MRI, failing to capture the intra-tumor temporal hemodynamic heterogeneity.

Therefore, to address the unmet clinical need for non-invasive ALN accurate prediction and bridge the technical gap in capturing spatiotemporal heterogeneity, this study proposes a novel radiomics framework leveraging voxel-wise TIC profile maps. Our approach integrates the quantification of both temporal hemodynamic patterns and their spatial distribution within primary breast tumors to predict ALN metastasis status preoperatively. In this study, a total of 615 breast cancer patients were retrospectively enrolled and randomly allocated into training and testing sets at 7:3 ratio. Based on wash-in rate, wash-out enhancement, and wash-out stability, each voxel within manually segmented 3D lesions that were categorized into 1 of 19 TIC subtypes from the DCE-MRI images. Three feature sets were derived from the manually segmented 3D lesions on the original phase-3 DCE-MR images (phase-3-radiomics set: radiomics features of third-phase DCE-MRI) and the newly generated TIC-profile-number 3D matrix images (type-19 set: composition ratio; type-19-radiomics set: radiomics features of TIC subtypes (type-19-radiomics). Student’s *t*-test and the least absolute shrinkage selection operator (LASSO) were used to select features. The selected features were used to develop four models (type-19, type-19-radiomics, type-19-combined (type-19 + type-19-radiomics), and phase-3-radiomics), respectively, by using a support vector machine (SVM) to predict ALN status. Model performance was assessed using sensitivity, specificity, accuracy, F1 score, and area under the curve (AUC).

## 2. Materials and Methods

### 2.1. Patients

**Figure 1:**
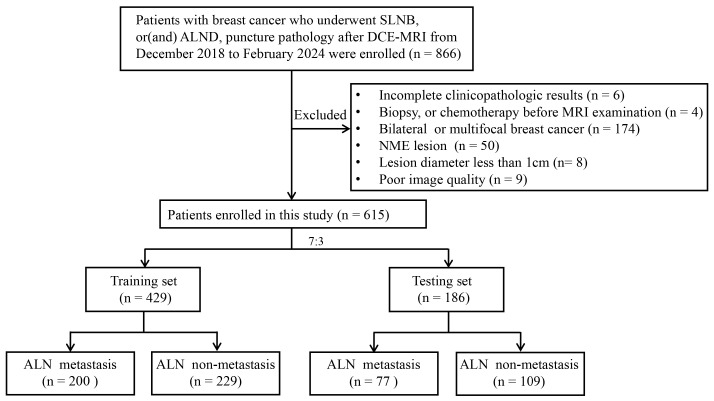
Flowchart of the study selection process. SLNB, sentinel lymph node biopsy; ALND, axillary lymph node dissection; NME, non-mass enhancement; ALN, axillary lymph node.

The study was approved by the corresponding hospitals’ Ethics Committees (KYKT2024-56-1), and patient informed consent was waived given its retrospective nature. We included 615 patients (age range, 25–83 years; mean age, 50 years ± 10.9 [SD]) with histologically confirmed breast cancer from our hospital between December 2018 and February 2024 (Table 1). Inclusion criteria were (1) histologically confirmed breast cancer; (2) MRI performed within 2 weeks before surgery; (3) ALN pathological results confirmed by ALND, puncture pathology, or SLNB and follow-up; and (4) satisfactory image quality. Exclusion criteria included (1) biopsy performed in the primary breast lesion, radiotherapy, or chemotherapy before MRI examination; (2) bilateral or multifocal breast cancer; (3) non-mass enhancement pattern presented on DCE-MRI; (4) lesion diameter less than 1 cm; and (5) incomplete or poor quality images. In this study, positive ALNs were determined by the results of ALND, SLNB, and puncture biopsy of lymph nodes, while negative ALNs were mainly determined by the results of ALND or SLNB and follow-up. The study’s flowchart is shown in Figure 1.

### 2.2. Clinical and Histopathology Data

Clinical and pathological data, including age, menstruation status, tumor size, histological type, histological grade, estrogen receptor (ER) status, progesterone receptor (PR) status, human epidermal growth factor receptor-2 (HER-2) status, and Ki-67 proliferation index were collected from the hospital electronic medical records. ALN metastasis was defined as macrometastasis (>2 mm) or micrometastasis (0.2–2 mm) in any lymph node [19]. The expression levels of ER, PR, HER-2, and Ki-67 were determined using immunohistochemistry (IHC), with positivity thresholds of 10% for ER or PR status, 14% for Ki-67, and HER-2 as negative (IHC 0/1+) and positive (IHC 3+ or FISH-confirmed IHC 2+) [20]. Breast cancers were grouped into Luminal, Luminal B, HER2-positive (HER2+), and triple-negative subtypes based on these markers.

### 2.3. MRI Data Collection

MRI scans were performed on a 3.0-T MRI system (Discovery MR750, GE Medical Systems, Milwaukee, WI, USA) with 8-channel breast coils in the prone position. DCE-MRI was performed using VIBRANT sequence (a three-dimensional T1WI technique) with the following parameters: repetition time (TR) = 4.5 ms, echo time (TE) = 2.1 ms, field of view = 360 × 360 mm, image matrix = 320 × 320, slice thickness = 1.4 mm with no gap, and flip angle = 12°. A total of nine phases, with one pre-contrast and eight postcontrast phases, were obtained. Each phase was acquired around 47 s, and the total scan time was about 7 min and 43 s. Gadoteric acid meglumine (Jiangsu Hengrui Pharmaceuticals Co., Ltd., Lianyungang, China) was administered intravenously with a flow rate of 2 mL/s at 0.1 mmol/kg of body weight using a power injector, followed by a 20 mL saline flush.

### 2.4. Tumor ROI Segmentation and Image Preprocessing

**Figure 2:**
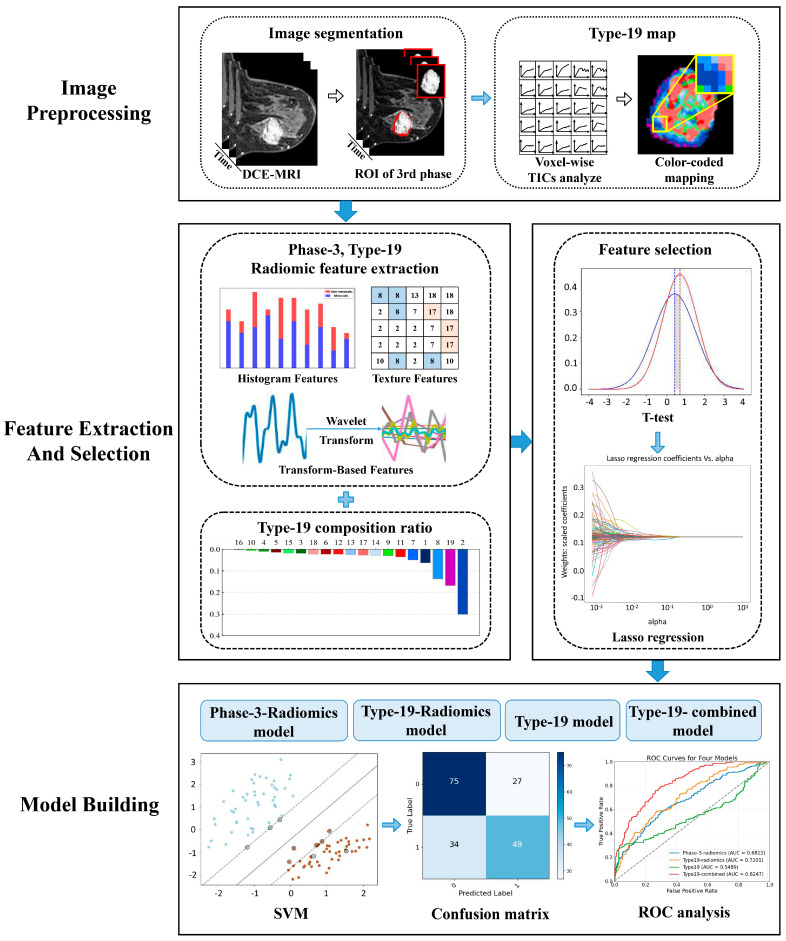
The workflow of this study.

Two junior radiologists (R.Y. and W.M.) with over 5 years of breast MRI experience manually segmented the tumor on the third phase of DCE-MR images, blinded to the clinical information of the patients. A senior radiologist (W.J.) with more than 10 years of breast MRI experience reviewed the delineation, with any discrepancies resolved by consensus. Voxel-by-voxel normalization of the post-contrast image (S) was conducted using the pre-contrast image (S0) as reference. Subsequently, a TIC profile for each voxel was generated for each segmented 3D lesion using an in-house Python (version 3.10) platform based on 19 TIC subtypes (see Figure 2).

### 2.5. Definition of Type-19

- Wash-in rate = $\frac{I_{p}\;-I_{0}}{I_{p}\;\times \;T_{p}}$, where I_0_ and I_p_ indicate the baseline and peak signal intensity, respectively, and T_P_ refers to the time used for the signal intensity to reach the peak within 3 phases. The wash-in rate of initial phase was divided into non-enhanced [<0.1], slow [0.1 to 0.5], medium [0.5 to 1.0], and fast [>1.0].
- Wash-out enhancement = $\frac{I_{last}\;-I_{p}}{I_{p}}$, where I_last_ and I_p_ refer to the peak signal intensity within 3 phases and signal intensity at the last phase, respectively. Wash-out enhancement is divided into persistent [>0.05], plateau [−0.05 to 0.05], and decline [<−0.05].
- Wash-out stability: The residual sum of squares (RSS) was used to calculate the degree in the oscillation of wash-out enhancement: wash−out stability = $\sqrt{\frac{\sum_{i=p}^{n}{\left(\frac{I_{i}-f\left(I_{i}\right)}{I_{0}}\right)}^{2}}{n}}$, where i ∈ {p,…, n}, p is the number of phases corresponding to T_P_ and n is the number of the last phase. I_i_ is the signal intensity in phase i, and f(I_i_) is the linear predicted signal intensity of I_i_. Wash-out stability is divided into steady [<0.1] and unsteady [≥0.1]).

**Figure 3:**
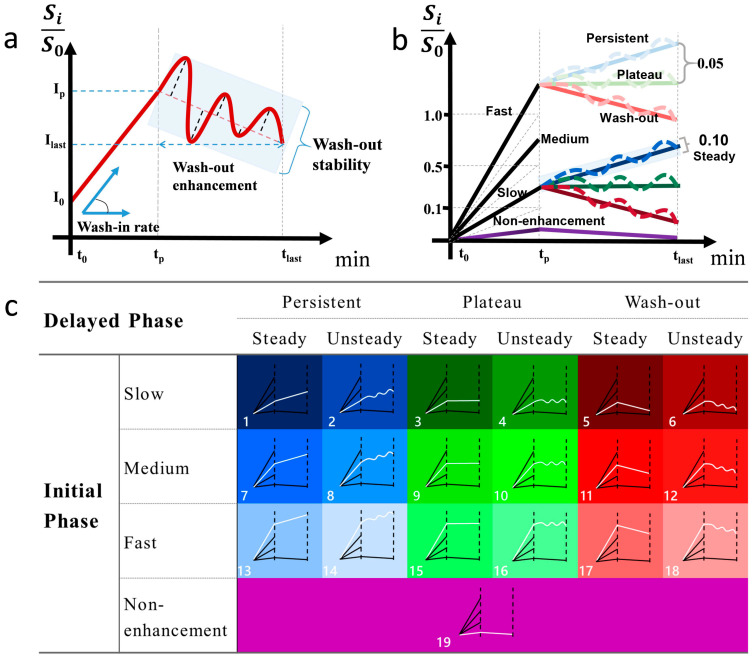
Definition and illustration of type-19 TIC profiles. The definition of the semiquantitative parameters (**a**). Cutoff value for wash-in rate, wash-out enhancement, and stability (**b**). Color-coded diagram for type-19 classification (**c**).

The TIC profiles were classed into 19 unique subtypes (3 × 3 × 2 + 1, where the “+1” represents the non-enhanced type of wash-in rate) based on the attributes of their wash-in rate (non-enhanced, slow, medium, and fast), wash-out enhancement (persistent, plateau, and decline), and wash-out stability (steady and non-steady). This categorization covers all possible types of TIC profile morphologies to ensure inclusivity (see Figure 3).

### 2.6. Feature Extraction

#### 2.6.1. Type-19 Feature Set

The TIC profile of each voxel within the 3D lesion was classified into one subtype of 19 defined TIC profiles voxel by voxel, and the percentage of voxels belonging to the same subtype in each breast lesion as a proportion of all voxels in the ROI is taken as a feature set (19 types).

#### 2.6.2. Phase-3-Radiomics Feature Set and Type-19-Radiomics Feature Set

A novel 3D matrix was constructed for each segmented lesion by assigning the corresponding voxel to its respective type-19 profile. Subsequently, two sets of radiomics features were extracted using the Pyradiomics package (version 3.1.0) on an in-house Python platform, with the extraction sources specified as follows: (1) Phase-3-radiomics: features extracted directly from the manually segmented 3D lesions on the original DCE-MR images; (2) type-19-radiomics: features extracted from the newly constructed 3D matrix of TIC-profile code. Each feature set comprised 1130 features, categorized into original (*n* = 107), log-transformed (*n* = 279), and wavelet features (*n* = 744). Original features included shape (*n* = 14), first-order (*n* = 18), and texture features (*n* = 75). Log-transformed features were calculated sigma values (1.0 mm, 3.0 mm, and 5.0 mm) applied to the first-order and texture features, yielding 279 features ((18 + 75) × 3 = 279). Wavelet features were derived from filtering combinations (2^3^ = 8) of high-pass and low-pass filters across three dimensions (x, y, z), resulting in 744 features (93 × 8 = 744).

#### 2.6.3. Type-19-Combined Feature Set

By combining the type-19-radiomics feature set with the type-19 feature set, the resulting type-19-combined feature set incorporates both the ratio of 19 subtypes per voxel within the breast lesion region and their corresponding radiomics features (1130 + 19 = 1149). Then, we selected the optimal features to construct the type-19 combined model.

### 2.7. Feature Selection and Ranking

A stratified sampling method was employed to partition the dataset into a training set and a validation set, with a ratio of 7:3. Initially, a Student’s *t*-test was performed on two independent samples to identify features that exhibited statistically significant differences (*p* < 0.05) between the lymph node metastatic group and the non-metastatic group. Subsequently, we employed the least absolute shrinkage and selection operator (LASSO) regression algorithm for feature selection, overfitting prevention, and increasing interpretability. Features with zero regression coefficients after shrinkage will be excluded from the next step of model construction.

### 2.8. Model Construction

Four models were developed using the support vector machine (SVM), with each model trained using a distinct set of selected features. The specific four models were defined as follows: (1) Phase-3-radiomics model: Trained and developed using the selected phase-3-radiomics features extracted from manually segmented 3D lesions on original phase-3 DCE-MR images. (2) Type-19 model: Trained and developed using the selected type-19 features that represent the ratio of each of the 19 TIC subtypes per voxel within breast lesion regions. (3) Type-19-radiomics model: Trained and developed using the selected type-19-radiomics features extracted from the newly generated 3D matrix of TIC-profile numbers. (4) Type-19-combined model: Trained and developed using the combined set of selected type-19 features and selected type-19-radiomics features. A 10-fold cross-validation scheme was used on the training set, and independent testing was performed on the test set to validate the model’s performance.

### 2.9. Statistical Analysis

We performed statistical analyses using SPSS software (version 26.0) and R software (Version 4.3.1). Quantitative variables were compared using Student’s *t*-test or the Mann–Whitney U test, while qualitative variables were compared using the chi-square test or Fisher’s test. Model performance was assessed by calculating the area under the ROC (AUC), accuracy, specificity, sensitivity, precision, and F1 score as well. The comparison of ROC curves was performed by the DeLong test. A two-sided *p* value less than 0.05 was considered statistically significant.

## 3. Results

### 3.1. Clinicopathologic Characteristics

A total of 615 patients were enrolled based on the inclusion and exclusion criteria, consisting of 277 ALN metastasis and 338 ALN non-metastasis patients. Patients were randomly divided into training (*n* = 430) and testing sets (*n* = 185) at a ratio of 7:3. The clinicopathologic data of the patients are summarized in Table 1.

### 3.2. Feature Extraction and Selection

#### 3.2.1. Type-19 Feature Set

**Figure 4:**
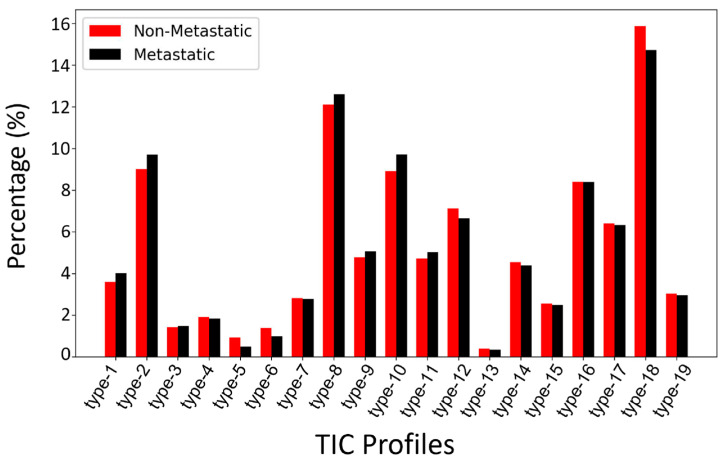
Comparison of type-19 TIC profile composition ratios between breast cancer with ALN metastasis and those without ALN metastasis across all subjects.

As shown in Figure 4, both the type-19 composition ratios of breast cancer lesions with ALN metastasis (*n* = 277) and those without ALN metastasis (*n* = 338) were very similar, both with 18th (14.7% vs. 15.9%), 8th (12.6% vs. 12.1%), 10th (9.7% vs. 8.9%), 2nd (9.7% vs. 9.0%), and 16th (8.3% vs. 8.4%) as the top 5 TIC profiles, together accounting for more than half of all TIC profiles. In terms of wash-in rate, in both the ALN metastasis group and the non-metastasis group, the most frequent TIC subtype was medium wash-in (41.9% vs. 40.5%), followed by fast wash-in (36.7% vs. 38.2%) and slow wash-in (18.5% vs. 18.3%), while for wash-out enhancement, the most frequent TIC subtype was decline wash-out (34.2% vs. 36.5%), followed by persistent (33.8% vs. 32.5%) and plateau (29.0% vs. 28.0%). The 5th TIC subtype was statistically different between the positive ALN and negative ALN group (0.5% vs. 0.9%, *p* = 0.046), and none of the other 18 TIC subtypes were statistically different between the two groups.

#### 3.2.2. Radiomics Feature Selection and Ranking

**Figure 5:**
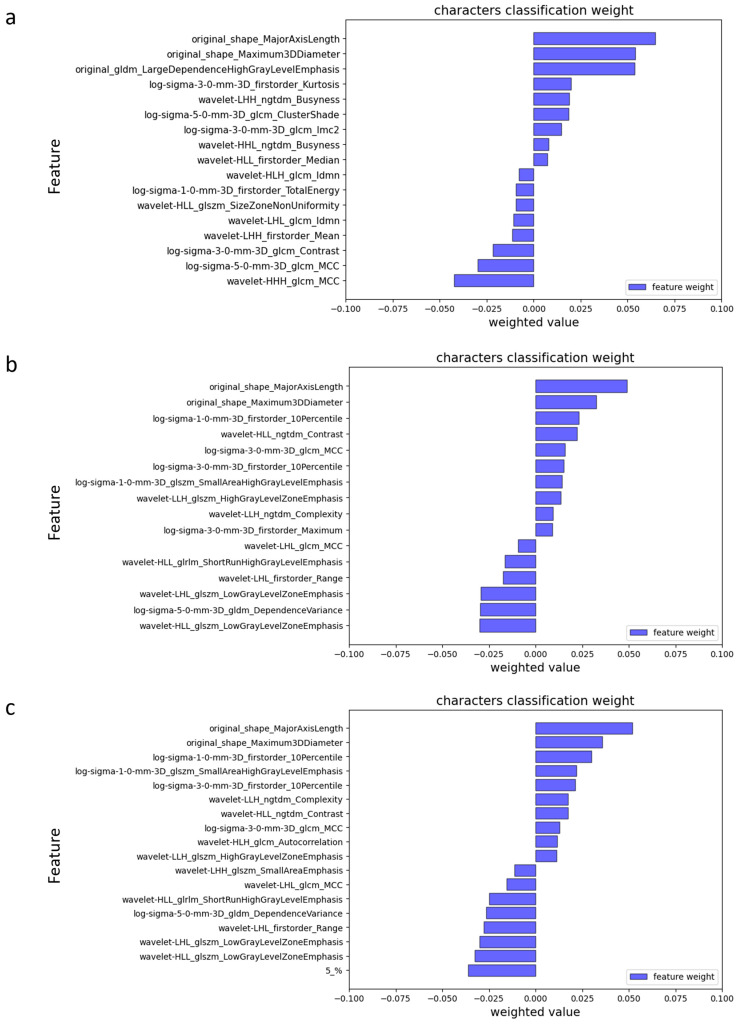
Feature importance ranking based on Lasso coefficients in phase-3-radiomics (**a**), type-19-radiomics (**b**), and type-19-combined (**c**).

**Figure 6:**
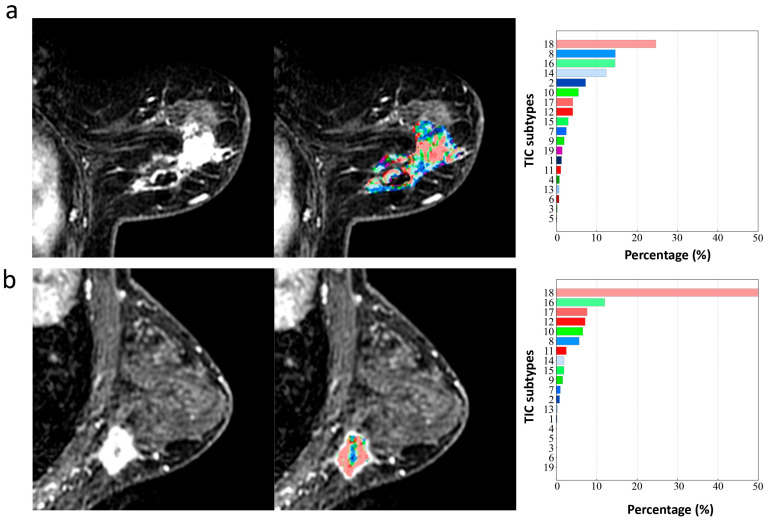
The type-19 color-coded maps of two breast cancer patients with ALN metastasis (**a**) and without ALN metastasis (**b**). All the 19 TIC subtypes occurred in the primary lesion of breast cancer with ALN metastasis with uneven distribution (**a**), while only 15 TIC subtypes occurred in the primary lesion of breast cancer without ALN metastasis, and were predominant in TIC 18 with more uniform distribution (**b**). The value of extracted texture features based on the type-19 color-coded map in the ALN metastasis group (**a**) are greater than those in the ALN non-metastasis group (**b**) (e.g., log-sigma-5-0-mm-3D_glrlm_RunVariance, 27.20 vs. 21.88; log-sigma-5-0-mm-3D_gldm_DependenceVariance, 30.33 vs. 21.88; wavelet-LLL _glrlm_ ShortRun HighGrayLevelEmphasis, 3.83 vs. 2.54; wavelet-HHL _firstorder_Variance, 3.60 vs. 2.32).

Using Student’s *t*-test, 677, 255, and 679 features were selected from the type-19-radiomics feature set, the phase-3-radomics feature set, and type-19-combined feature set, respectively, and then 16, 17, and 18 features were finally selected as the optimal features using the LASSO method. The selected type-19-radiomics features contained 2 shape, 4 first-order, and 10 texture features (Figure 5a). The selected phase-3-radiomics features included 2 shape, 4 first-order, and 11 texture features (Figure 5b). The selected type-19-combined features included 2 shape, 4 first-order, 11 texture, and 1 type-19 features (Figure 5c). The top 5 features with the highest absolute coefficient included 3 texture features and 2 shape features for the type-19-radiomics feature set (Figure 5a), 2 texture, 2 shape, and 1 first-order feature for the phase-3-radiomics feature set (Figure 5b), and 3 texture, 1 shape, and 1 first-order feature for the type-19-combined feature set (Figure 5c). The breast cancer with ALN metastasis exhibited greater texture heterogeneity in the type-19 color-coded map than those without ALN metastasis (Figure 6).

### 3.3. Model Performance

**Figure 7:**
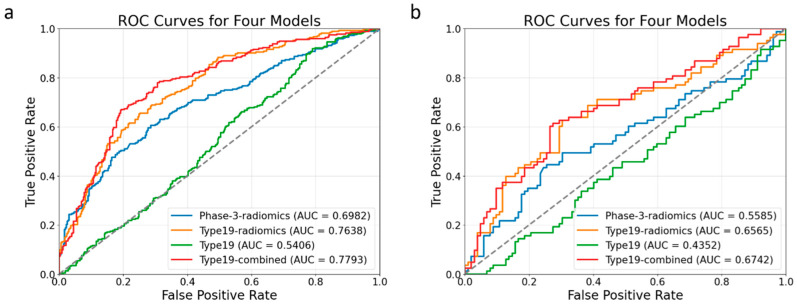
ROC curves of four models in the 10-fold cross-validation set (**a**) and the independent testing set (**b**).

Among four models, in both 10-fold cross-validation (Figure 7a) and the independent testing set (Figure 7b), the type-19-combined model achieved the highest AUC (0.779, 0.674), followed by type-19-radiomics model (0.764, 0.657) and phase-3-radiomics model (0.698, 0.559), and the type-19 model (0.541, 0.435) (Figure 7, Table 2). The type-19-combined model significantly outperformed the phase-3-radiomics model (AUC = 0.779 vs. 0.698, *p* < 0.001; AUC = 0.674 vs. 0.559, *p* = 0.006) and type-19 model (AUC = 0. 779 vs. 0.541, *p* < 0.001; AUC = 0.674 vs. 0.435, *p* < 0.001) in both 10-fold cross-validation and independent testing set (Table 2 and Table 3). However, the type-19-combined model showed insignificantly better performance values than the type-19-radiomics model in both the 10-fold cross-validation set (AUC = 0.779 vs. 0.764, *p* = 0.156) and the independent testing set (AUC = 0.674 vs. 0.657, *p* = 0.522) (Table 2 and Table 3). The type-19-radiomics model outperformed the phase-3-radiomics model (AUC = 0.764 vs. 0.698, *p* = 0.002; AUC = 0.657 vs. 0.559, *p* < 0.001) and type-19 model (AUC = 0.720 vs. 0.549, *p* < 0.001, AUC = 0.704 vs. 0.452, *p* = 0.036) in both 10-fold cross-validation and the independent testing set. (Table 2 and Table 3). The phase-3-radiomics performed better than the type-19 model (AUC = 0.698 vs. 0.540, *p* < 0.001, AUC = 0.559 vs. 0.435, *p* = 0.028) in both the 10-fold cross-validation and independent testing sets.

## 4. Discussion

This study developed and validated a radiomics model using the DCE-MRI TIC profile map to predict ALN metastasis in breast cancer. The type-19-combined (type-19 + type-19-radiomics) model and type-19-radiomics significantly outperformed the conventional phase-3-radiomics model, highlighting the novel approach’s potential in predicting ALN metastatic status by quantifying temporal and spatial hemodynamic heterogeneity simultaneously through radiomics analysis based on the TIC profile map.

In recent years, radiomics, which can extract quantitative features from medical images, has been extensively applied in breast cancer research for predicting ALN metastatic status [19,20]. Our study revealed that texture features were most predictive among all the extracted radiomics features from the third phase of DCE in predicting the ALN metastasis, suggesting that the spatial heterogeneity of the tumor contributes most to predicting ALN metastasis, which is consistent with previous studies [21,22]. Both Luo et al. [21] and Liu et al. [22] demonstrated that the texture features contributed most to the prediction of ALN metastatic status. However, our phase-3-radiomics model yielded suboptimal predictive performance in both 10-fold cross-validation and independent testing sets, with an AUC of 0.698 and 0.559, respectively. This may be because the phase-3-radiomics model developed by extracting radiomic features from a single phase only exploited spatial hemodynamic heterogeneity without accounting for the temporal hemodynamic heterogeneity. Notably, most current radiomics-related models for predicting ALN metastasis rely on single-phase radiomics [22,23,24], lacking the integration of dynamic temporal features. Thus, it is necessary to develop a novel method that can simultaneously quantify the spatial and temporal heterogeneity of tumors to further improve the efficacy of predicting ALN metastasis.

In our study, we integrated radiomics analysis with a model-free and data-driven method of mapping voxel-wise TIC profiles subtypes within the 3D whole tumor, aiming to simultaneously quantify temporal and spatial hemodynamic heterogeneity. We demonstrated that both type-19-combined and type-19-radiomics models significantly outperformed the phase-3-radiomics and type-19 models in the testing set in predicting the ALN metastasis, underscoring the robustness of this novel approach. Our findings align with previous related studies. For example, Shan et al. showed superior performance of a combined radiomics and kinetic curve pattern model in distinguishing ALN metastatic status in breast cancer [25]. Liu et al. presented that the model incorporating radiomics features and pharmacokinetic parameters had higher prediction ability than any single model for the preoperative evaluation of SLN metastasis in breast cancer [26]. Luo et al. extracted radiomic features from PK-DCE-MRI images of axillary lymph nodes and found them to be more helpful than pharmacokinetic quantitative features for diagnosis of metastatic axillary nodes [21]. In our study, owing to the combination of spatial and temporal hemodynamic heterogeneity, the type-19-radiomics and type-19-combined model showed enhanced efficacy compared to the phase-3-radiomics model or type-19 model alone. Notably, texture features were predominant in the selected type-19-radiomics feature set, consistent with Liu et al.’s findings [27] that texture features extracted from wash-out, wash-in, or signal enhancement ratio maps based on DCE-MRI images demonstrated significant value in predicting ALN metastasis. And we found the texture heterogeneity in the type-19 color-coded map was greater in breast cancer patients with ALN metastasis than those without ALN metastasis. Previous studies have indicated that the underlying microvasculature heterogeneity is a major driver of this inter-tumor and intra-tumor microenvironment heterogeneity and contribute to ALN metastasis in breast cancer [28]. Specifically, primary breast tumors with ALN metastasis usually have a more disorganized tumor vascular network, which makes it easier for tumor cells to escape into the lymphatic system. The unevenly distributed microvessels (e.g., areas of vascular proliferation adjacent to avascular necrotic regions) of breast cancer with ALN metastasis, leading to the hemodynamic distribution of the heterogeneous contrast agent, and the corresponding uneven distribution of TIC subtypes voxel by voxel. Such larger differences in TIC subtypes are visualized in the type-19 color-coded map as a more chaotic distribution of the 19 colors—that is, higher texture heterogeneity. In contrast, tumors without ALN metastasis generally have more uniform spatial hemodynamics, as their vascular networks are relatively well-organized, reducing the likelihood of cells escaping into the lymphatic system, leading to a more uniform distribution of TIC subtypes and lower texture heterogeneity [29,30]. Therefore, the novel noninvasive tool we developed in this study that can quantify and visualize the temporal and spatial microvascular heterogeneity of primary tumors can better predict ALN metastatic status in breast cancer.

In our study, the type-19-combined model achieved the highest AUC, accuracy, precision, and F1 score in both the 10-fold cross-validation and the independent testing, yet its advantage over the type-19-radiomics model was not statistically significant, implying that the type-19-radiomics features predominantly contribute to its performance. This is evidenced by the nearly identical proportions of the 19 TIC profiles between the ALN metastatic and non-metastatic groups, contrasting with prior findings that malignant and benign breast lesions showed vastly different composition ratios of 19 TIC profiles [13]. In addition, our previous work showed that type-19 composition ratios underperformed in discriminating the histological grades (high grade vs. low grade) and proliferation status (high Ki-67 index vs. low Ki-67 index) of malignant lesions and demonstrated worse performance in classifying four molecular subtypes (Luminal A, Luminal B, HER-2, and triple-negative) of breast cancer. This all indicates that breast cancer shares similar TIC profile distributions, which makes it a much more challenging task to further classify their subtypes solely based on the composition ratios of TIC profiles. This aligns with a previous study showing no significant kinetic-parameter differences in primary breast cancer lesions between patients with and without ALN metastasis [31]. By contrast, a number of previous studies [21,32], including our results, all confirmed that radiomics features extracted from even a single phase of DCE-MRI reliably predict ALN status in breast cancer, underscoring the importance of spatial heterogeneity. Furthermore, the superior performance of the type-19-radiomics model over the phase-3-radiomics model demonstrates the integrating temporal and spatial hemodynamic heterogeneity through TIC-based radiomics adds meaningful information and may enhance clinical utility.

The study encountered several limitations. Firstly, it was a retrospective analysis conducted at a single institution, necessitating future external validation of the model’s stability and clinical applicability using a multi-center dataset. Secondly, the manual segmentation of the tumor was time-consuming and subject to inter-observer variability. An automatic segmentation model with higher accuracy and stability would be highly beneficial. Thirdly, radiomics features were not directly extracted from ALN MRI images due to the complexities involved in matching the lymph node identified in pathology with those visible in MR images and the fact that breast MRI scans may not comprehensively cover all ALNs in certain cases. Lastly, the sensitivity and specificity of the type-19-radiomics model and the type-19-combined model are relatively low and need to be greatly improved by further study in the future.

## 5. Conclusions

In conclusion, this study developed a novel DCE-MRI-based radiomics model using TIC profile maps to predict ALN metastasis in breast cancer. The type-19-radiomics model, which integrates temporal hemodynamics (TIC profiles) and spatial heterogeneity (radiomics features), significantly outperformed conventional single-phase radiomics models. Texture features were found to be the most predictive, highlighting the importance of spatial heterogeneity. While the combined model achieved the highest metrics, its advantage over the type-19-radiomics model was not statistically significant, indicating radiomics features are the main contributors to the performance. This integrated spatiotemporal analysis addresses a key limitation of previous single-phase approaches. Overall, this study introduces a promising noninvasive imaging biomarker for the preoperative prediction of breast cancer ALN metastasis. This tool has the potential to optimize clinical decision-making by providing accurate, individualized ALN status assessments, thereby supporting the customization of treatment strategies, such as by avoiding unnecessary axillary lymph node dissection in patients with low metastasis risk and ultimately improving the management of breast cancer patients.

## Figures and Tables

**Table 1:** Clinicopathologic characteristics of patients.

Characteristic	ALN Metastasis (*n* = 277)	ALN Non-Metastasis (*n* = 338)
Age, (mean ± SD), year	51.13 ± 10.63	50.14 ± 11.13
Menopausal status, *n* (%)		
Premenopause	117 (42.2%)	172 (50.9%)
Postmenopause	160 (57.8%)	166 (49.1%)
Tumor size, (mean ± SD), cm	3.20 ± 1.66	2.42 ± 1.22
Histological type, *n* (%)		
Invasive ductal carcinoma	264 (95.3%)	287 (84.9%)
Invasive lobular carcinoma	6 (2.2%)	8 (2.4%)
Others	7 (2.5%)	43 (12.7%)
Histological grade, *n* (%)		
Grade I	8 (2.9%)	18 (5.3%)
Grade II	112 (40.4%)	132 (39.1%)
Grade III	143 (51.6%)	147 (43.5%)
Not available	14 (5.1%)	41 (12.1%)
ER status		
Negative	78 (28.2%)	94 (27.8%)
Positive	199 (71.8%)	244 (72.2%)
PR status		
Negative	93 (33.6%)	124 (36.7%)
Positive	184 (66.4%)	214 (63.3%)
HER-2 status		
Negative	174 (62.8%)	250 (74.0%)
Positive	103 (37.2%)	88 (26.0%)
Ki-67 status		
<14	33 (11.9%)	50 (14.8%)
≥14	244 (88.1%)	288 (85.2%)
Molecular subtypes		
Luminal A	16 (5.8%)	33 (9.8%)
Luminal B	192 (69.3%)	210 (62.1%)
HER-2 positive	42 (15.2%)	39 (11.5%)
Triple negative	27 (9.7%)	56 (16.6%)

SD, standard deviation; ER, estrogen receptor; PR, progesterone receptor; HER-2, human epidermal growth factor receptor-2.

**Table 2:** The model’s performance in 10-fold cross-validation and independent testing.

		Accuracy	Sensitivity	Specificity	Precision	F1 Score	AUC
10-fold cross-validation set	Phase-3-radiomics	0.6527	0.5512	0.7375	0.6462	0.5883	0.6982
	Type-19-radiomics	0.6930	0.6926	0.6950	0.6573	0.6689	0.7638
	Type-19	0.5178	0.2860	0.7071	0.4259	0.3375	0.5406
	Type-19-combined	0.7332	0.6621	0.7933	0.7379	0.6881	0.7793
Independent testing set	Phase-3-radiomics	0.6186	0.4337	0.7549	0.5902	0.5000	0.5585
	Type-19-radiomics	0.6595	0.6024	0.7059	0.6250	0.6135	0.6565
	Type-19	0.4811	0.2530	0.6667	0.3138	0.3043	0.4352
	Type-19-combined	0.6703	0.5904	0.7353	0.6447	0.6164	0.6742

**Table 3:** DeLong test results on comparing four models.

	10-Fold Cross-Validation	Independent Testing Set
Type-19-radiomics vs. phase-3-radiomics	*p* = 0.002	*p* = 0.037
Type-19-radiomics vs. type-19	*p* < 0.001	*p* < 0.001
Type-19-combined vs. phase-3-radiomics	*p* < 0.001	*p* = 0.006
Type-19-combined vs. type-19	*p* < 0.001	*p* < 0.001
Type-19-combined vs. type-19-radiomics	*p* = 0.156	*p* = 0.522
Type-19 vs. phase-3-radiomics	*p* < 0.001	*p* = 0.028

## Data Availability

The data presented in this study are not publicly available due to privacy or ethical restrictions, but available on request from the corresponding author.

## Abbreviations

The following abbreviations are used in this manuscript:

DCE-MRI	Dynamic contrast-enhanced magnetic resonance imaging
TIC	Time-intensity curve
ALN	Axillary lymph node
ALND	Axillary lymph node dissection
SLNB	Sentinel lymph node biopsy
TR	Repetition time
TE	Echo time
AUC	Area under the curve
ROC	Receiver operating characteristic

## References

[1] Bray F., Laversanne M., Sung H., Ferlay J., Siegel R.L., Soerjomataram I., Jemal A. (2024). Global cancer statistics 2022: GLOBOCAN estimates of incidence and mortality worldwide for 36 cancers in 185 countries. CA Cancer J. Clin..

[2] Danko M.E., Bennett K.M., Zhai J., Marks J.R., Olson J.A.J. (2010). Improved staging in node-positive breast cancer patients using lymph node ratio: Results in 1788 patients with long-term follow-up. J. Am. Coll. Surg..

[3] Ahmed M., Purushotham A.D., Douek M. (2014). Novel techniques for sentinel lymph node biopsy in breast cancer: A systematic review. Lancet Oncol..

[4] Weigel M.T., Dowsett M. (2010). Current and emerging biomarkers in breast cancer: Prognosis and prediction. Endocr. Relat. Cancer.

[5] Langer I., Guller U., Berclaz G., Koechli O.R., Schaer G., Fehr M.K., Hess T., Oertli D., Bronz L., Schnarwyler B. (2007). Morbidity of sentinel lymph node biopsy (SLN) alone versus SLN and completion axillary lymph node dissection after breast cancer surgery: A prospective Swiss multicenter study on 659 patients. Ann. Surg..

[6] Schrenk P., Rieger R., Shamiyeh A., Wayand W. (2000). Morbidity following sentinel lymph node biopsy versus axillary lymph node dissection for patients with breast carcinoma. Cancer.

[7] Pesek S., Ashikaga T., Krag L.E., Krag D. (2012). The false-negative rate of sentinel node biopsy in patients with breast cancer: A meta-analysis. World J. Surg..

[8] Choi E.J., Youk J.H., Choi H., Song J.S. (2020). Dynamic contrast-enhanced and diffusion-weighted MRI of invasive breast cancer for the prediction of sentinel lymph node status. J. Magn. Reson. Imaging.

[9] Ya G., Wen F., Xing-Ru L., Zhuan-Zhuan G., Jun-Qiang L. (2022). Difference of DCE-MRI Parameters at Different Time Points and Their Predictive Value for Axillary Lymph Node Metastasis of Breast Cancer. Acad. Radiol..

[10] Goto M., Ito H., Akazawa K., Kubota T., Kizu O., Yamada K., Nishimura T. (2007). Diagnosis of breast tumors by contrast-enhanced MR imaging: Comparison between the diagnostic performance of dynamic enhancement patterns and morphologic features. J. Magn. Reson. Imaging.

[11] Liu H.-L., Zong M., Wei H., Lou J.-J., Wang S.-Q., Zou Q.-G., Shi H.-B., Jiang Y.-N. (2018). Differentiation between malignant and benign breast masses: Combination of semi-quantitative analysis on DCE-MRI and histogram analysis of ADC maps. Clin. Radiol..

[12] Winfield J.M., Payne G.S., Weller A., deSouza N.M. (2016). DCE-MRI, DW-MRI, and MRS in Cancer: Challenges and Advantages of Implementing Qualitative and Quantitative Multi-parametric Imaging in the Clinic. Top. Magn. Reson. Imaging.

[13] Liu Z., Yao B., Wen J., Wang M., Ren Y., Chen Y., Hu Z., Li Y., Liang D., Liu X. (2024). Voxel-wise mapping of DCE-MRI time-intensity-curve profiles enables visualizing and quantifying hemodynamic heterogeneity in breast lesions. Eur. Radiol..

[14] Gillies R.J., Kinahan P.E., Hricak H. (2016). Radiomics: Images Are More than Pictures, They Are Data. Radiology.

[15] Lambin P., Leijenaar R.T.H., Deist T.M., Peerlings J., de Jong E.E.C., van Timmeren J., Sanduleanu S., Larue R.T.H.M., Even A.J.G., Jochems A. (2017). Radiomics: The bridge between medical imaging and personalized medicine. Nat. Rev. Clin. Oncol..

[16] Song D., Yang F., Zhang Y., Guo Y., Qu Y., Zhang X., Zhu Y., Cui S. (2022). Dynamic contrast-enhanced MRI radiomics nomogram for predicting axillary lymph node metastasis in breast cancer. Cancer Imaging.

[17] Mao N., Dai Y., Lin F., Ma H., Duan S., Xie H., Zhao W., Hong N. (2020). Radiomics Nomogram of DCE-MRI for the Prediction of Axillary Lymph Node Metastasis in Breast Cancer. Front. Oncol..

[18] Chen H., Lan X., Yu T., Li L., Tang S., Liu S., Jiang F., Wang L., Huang Y., Cao Y. (2022). Development and validation of a radiogenomics model to predict axillary lymph node metastasis in breast cancer integrating MRI with transcriptome data: A multicohort study. Front. Oncol..

[19] Tagliafico A.S., Piana M., Schenone D., Lai R., Massone A.M., Houssami N. (2020). Overview of radiomics in breast cancer diagnosis and prognostication. Breast.

[20] Han X., Gong Z., Guo Y., Tang W., Wei X. (2024). Exploration of a noninvasive radiomics classifier for breast cancer tumor microenvironment categorization and prognostic outcome prediction. Eur. J. Radiol..

[21] Luo H.-B., Liu Y.-Y., Wang C.-H., Qing H.-M., Wang M., Zhang X., Chen X.-Y., Xu G.-H., Zhou P., Ren J. (2021). Radiomic features of axillary lymph nodes based on pharmacokinetic modeling DCE-MRI allow preoperative diagnosis of their metastatic status in breast cancer. PLoS ONE.

[22] Liu J., Sun D., Chen L., Fang Z., Song W., Guo D., Ni T., Liu C., Feng L., Xia Y. (2019). Radiomics Analysis of Dynamic Contrast-Enhanced Magnetic Resonance Imaging for the Prediction of Sentinel Lymph Node Metastasis in Breast Cancer. Front. Oncol..

[23] Chen Y., Li J., Zhang J., Yu Z., Jiang H. (2024). Radiomic Nomogram for Predicting Axillary Lymph Node Metastasis in Patients with Breast Cancer. Acad. Radiol..

[24] Wang C., Chen X., Luo H., Liu Y., Meng R., Wang M., Liu S., Xu G., Ren J., Zhou P. (2021). Development and Internal Validation of a Preoperative Prediction Model for Sentinel Lymph Node Status in Breast Cancer: Combining Radiomics Signature and Clinical Factors. Front. Oncol..

[25] Shan Y.-N., Xu W., Wang R., Wang W., Pang P.-P., Shen Q.-J. (2020). A Nomogram Combined Radiomics and Kinetic Curve Pattern as Imaging Biomarker for Detecting Metastatic Axillary Lymph Node in Invasive Breast Cancer. Front. Oncol..

[26] Liu M., Mao N., Ma H., Dong J., Zhang K., Che K., Duan S., Zhang X., Shi Y., Xie H. (2020). Pharmacokinetic parameters and radiomics model based on dynamic contrast enhanced MRI for the preoperative prediction of sentinel lymph node metastasis in breast cancer. Cancer Imaging.

[27] Liu C., Ding J., Spuhler K., Gao Y., Serrano Sosa M., Moriarty M., Hussain S., He X., Liang C., Huang C. (2019). Preoperative prediction of sentinel lymph node metastasis in breast cancer by radiomic signatures from dynamic contrast-enhanced MRI. J. Magn. Reson. Imaging.

[28] Arneth B. (2019). Tumor Microenvironment. Medicina.

[29] Liu Y., Li X., Zhu L., Zhao Z., Wang T., Zhang X., Cai B., Li L., Ma M., Ma X. (2022). Preoperative Prediction of Axillary Lymph Node Metastasis in Breast Cancer Based on Intratumoral and Peritumoral DCE-MRI Radiomics Nomogram. Contrast Media Mol. Imaging.

[30] Junttila M.R., de Sauvage F.J. (2013). Influence of tumour micro-environment heterogeneity on therapeutic response. Nature.

[31] Dong X., Chunrong Y., Hongjun H., Xuexi Z. (2019). Differentiating the lymph node metastasis of breast cancer through dynamic contrast-enhanced magnetic resonance imaging. BJR Open.

[32] Schacht D.V., Drukker K., Pak I., Abe H., Giger M.L. (2015). Using quantitative image analysis to classify axillary lymph nodes on breast MRI: A new application for the Z 0011 Era. Eur. J. Radiol..

